# Removal of the Water‐Soluble Plastic Pollutant Poly(vinyl alcohol) Using Kaolinite: Physicochemical Effects and Adsorption Mechanisms

**DOI:** 10.1002/open.70331

**Published:** 2026-11-05

**Authors:** Gul Abad Afridi, Tasleem Hussain Shah, Muhammad Fakhar‐e‐Alam, Mehwish Huma, Muhammad Zakria, Muhammad Imran, Muhammad Ismail, Abbas Nasir, Qaisar Abbas Bhatti, Sakil Mahmud

**Affiliations:** ^1^ Department of Chemistry Kohat University of Science and Information Technology Kohat Pakistan; ^2^ Department of Physics Government College University Faisalabad Pakistan; ^3^ Department of Chemical and Life Sciences Qurtuba University of Science and Information Technology Dera Ismail Khan Khyber Pakhtunkhwa Pakistan; ^4^ National Institute of Lasers and Optronics (NILOP)‐College Pakistan Institute of Engineering and Applied Sciences (PIEAS)‐University Islamabad Pakistan; ^5^ Research Center for Advanced Materials Science King Khalid University Abha Saudi Arabia; ^6^ Department of Physics Abdul Wali Khan University Mardan Mardan Khyber Pakhtunkhwa Pakistan; ^7^ School of Physics and Materials Sciences Guangzhou University Guangzhou China; ^8^ Department of Textile Engineering Faculty of Engineering Daffodil International University Dhaka People’s Republic of Bangladesh; ^9^ Ivory V. Nelson Center for the Sciences Department of Chemistry and Physics Lincoln University Oxford Pennsylvania USA

**Keywords:** adsorption mechanism, kaolinite clay, poly(vinyl alcohol) adsorption, polymer/clay interactions, water‐soluble plastics

## Abstract

The adsorption behavior of poly(vinyl alcohol) (PVOH), a representative water‐soluble plastic, onto kaolinite clay was systematically investigated under varying physicochemical conditions. Four PVOH fractions with molecular masses ranging from 49 to 125 × 10^3^ g mol^–1^ were evaluated to determine the effects of pH, electrolyte concentration, thermal treatment, homogenization, and ultrasonication on adsorption performance. Adsorption equilibrium and kinetic analyses showed that the Langmuir isotherm (*R* ^2^ > 0.99) and pseudo‐second‐order kinetic model (*R* ^2^ > 0.99584) best described the adsorption process. Adsorption capacity increased with polymer molecular mass and decreased by approximately 18% as pH increased from 2 to 10 due to increasing negative surface charge and electrostatic repulsion. Elevated electrolyte concentrations reduced adsorption through ionic competition and active‐site blockage. In contrast, ultrasonication and thermal pretreatment significantly enhanced adsorption by increasing surface area and surface charge density. Spectroscopic and physicochemical analyses confirmed that hydrogen bonding between PVOH hydroxyl groups and kaolinite silanol/aluminol groups, together with van der Waals interactions, dominated the adsorption mechanism. These findings demonstrate the potential of modified kaolinite as a sustainable and low‐cost adsorbent for mitigating soluble plastic pollution.

## Graphical abstract

Kaolinite provides a low‐cost adsorbent for removing water‐soluble poly(vinyl alcohol) from aqueous solutions. Adsorption increases with polymer molecular mass and is enhanced by moderate thermal pretreatment and ultrasonication, while increasing pH and electrolyte concentration suppress uptake. Hydrogen bonding and van der Waals interactions contribute to polymer–clay association, highlighting the potential of kaolinite for soluble polymer pollution mitigation.

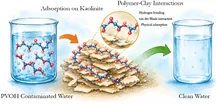

## 1 Introduction

Plastic pollution has emerged as one of the most critical environmental challenges of the twenty‐first century; however, research and regulatory attention have predominantly focused on visible macroplastics and insoluble microplastics, while the environmental fate of water‐soluble polymers remains comparatively underexplored. Unlike conventional particulate plastics, soluble polymeric contaminants dissolve completely in aqueous systems, allowing them to bypass traditional filtration and wastewater treatment processes and disperse rapidly through aquatic and terrestrial environments [1]. Their molecular‐scale dispersion not only complicates detection and removal but also enhances their interaction with dissolved contaminants, mineral surfaces, and biological systems. Recent studies have demonstrated that soluble polymers can alter water chemistry, interfere with microbial and metabolic pathways, and facilitate the transport of hazardous cocontaminants, including antibiotics, heavy metals, and persistent organic pollutants, thereby increasing ecological and toxicological risks [2]. Consequently, understanding the environmental behavior of water‐soluble plastics has become increasingly important for sustainable pollution management and water remediation technologies.

Among water‐soluble polymers, poly(vinyl alcohol) (PVOH) has attracted considerable industrial and scientific interest because of its excellent film‐forming ability, chemical resistance, biodegradability, mechanical flexibility, and hydrophilic nature [3, 4, 5]. PVOH is widely used in the food, pharmaceutical, textile, paper, packaging, and agricultural industries, resulting in its continuous release into wastewater streams and natural ecosystems [6, 7, 8]. Although PVOH is often considered environmentally benign due to its water solubility, growing evidence suggests that dissolved polymeric materials may persist in aquatic environments and participate in complex interfacial interactions with naturally occurring minerals [9]. In natural systems, clay minerals are among the most reactive geochemical phases owing to their high specific surface area, layered structure, ion‐exchange capacity, and abundance of surface‐active functional groups [10, 11, 12]. Among these minerals, kaolinite is particularly important due to its widespread environmental occurrence, chemical stability, low toxicity, and strong adsorption capacity for organic and inorganic pollutants [13, 14, 15, 16]. Structurally, kaolinite is a 1:1 dioctahedral aluminosilicate consisting of alternating silica tetrahedral sheets and alumina octahedral sheets connected through hydrogen bonding [17]. The presence of surface silanol and aluminol groups gives kaolinite unique adsorption characteristics and enables strong interactions with hydroxyl‐containing polymers, such as PVOH.

Previous studies investigating polymer–clay interactions have primarily focused on adsorption capacity, nanocomposite fabrication, or basic spectroscopic characterization, while comparatively little attention has been given to the dynamic environmental factors that govern adsorption mechanisms [18, 19, 20]. In particular, the combined effects of pH, electrolyte concentration, polymer molecular mass, thermal pretreatment, homogenization, and ultrasonication on adsorption behavior remain poorly understood. Existing adsorption studies often assume idealized conditions and overlook how physicochemical modifications of clay surfaces alter adsorption energetics, colloidal stability, and polymer conformation. Furthermore, despite reports suggesting that hydrogen bonding plays a dominant role in PVOH adsorption, the relative contributions of electrostatic interactions, surface charge modification, and cavitation‐induced surface activation remain insufficiently clarified. Addressing these gaps is essential for developing realistic adsorption models and designing effective clay‐based remediation systems for soluble plastic contaminants. Therefore, the present study systematically investigates the adsorption behavior and adsorption mechanism of PVOH on kaolinite under varying physicochemical conditions, including pH, electrolyte concentration, molecular mass, thermal treatment, homogenization, and ultrasonication. By integrating adsorption isotherms, kinetic analysis, electrokinetic measurements, surface characterization, and spectroscopy, this work provides mechanistic insight into polymer–clay interactions and establishes the potential of modified kaolinite as a low‐cost, environmentally sustainable adsorbent for mitigating soluble plastic pollution.

## 2 Experimental Section

### 2.1 Materials

Kaolinite (China clay, Sigma‐Aldrich, Shanghai, China) with a fine particle size was employed as the adsorbent. Four PVOH fractions with average molecular masses of 49, 72, 96, and 125 kDa, denoted as PVOH_1_, PVOH_2_, PVOH_3_, and PVOH_4_, respectively, were obtained from BASF (Germany). Analytical‐grade nitric acid (HNO_3_), sodium hydroxide (NaOH), potassium hydroxide (KOH), sodium chloride (NaCl), and potassium chloride (KCl) were purchased from Sigma‐Aldrich (Shanghai, China) and used without further purification. Deionized distilled water with a conductivity of 2.5 ± 0.02 μS cm^–1^ and a pH of 6.5 ± 0.1 was used throughout all experiments.

PVOH stock solutions (1000 mg L^−1^) were prepared by dissolving the accurately weighed polymer powder in deionized water under continuous magnetic stirring at 80 °C for 2 h. After cooling to room temperature, the solutions were passed through a 0.45‐µm cellulose acetate membrane filter to remove any residual undissolved particles or microgel aggregates. Working solutions (10–80 mg L^−1^) were prepared by serial dilution immediately before the adsorption experiments.

### 2.2 Methods and Analytics

*Preparation and Thermal Treatment of Kaolinite*: The kaolinite samples were dried at 60 °C for 12 h before use and stored in airtight containers. Thermal treatment was performed by placing kaolinite in ceramic crucibles and heating them in a muffle furnace (SX3‐2.5‐12, China) at temperatures ranging from 298 to 523 K for 3 h. After heating, samples were cooled naturally to room temperature and stored in sealed glass bottles before use.

*Ultrasonication Treatment*: The effect of mechanical treatment was examined through ultrasonication. Kaolinite suspensions (10 wt%) were prepared in Millipore water and treated using a high‐intensity ultrasonic processor (UP200s, Dr. Hielscher GmbH, Germany) operating at 24 kHz with a maximum output power of 200 W. Sonication was performed for 5–30 min at room temperature.

*Surface Charge Density Measurements*: The surface charge density of kaolinite dispersions was measured by colloidal titration using a particle charge detector (PCD‐04, Mütek GmbH, Germany). Polydiallyldimethylammonium chloride (PDADMAC) and sodium polyethylene sulfonate (PES‐Na) were employed as titrants for cationic and anionic systems, respectively. The surface charge density (q) was calculated using Equation (1), where *C*_titrant_ represents titrant concentration (meq L^–1^), *V* is the sample volume (L), *V*_titrant_ is the equivalent titrant volume (L), and *m* denotes the sample concentration (g L^–1^).

(1) $$\begin{array}{cc} & q=\frac{C_{\text{titrant}}\;\times \;V_{\text{titrant}}}{V\;\times \;m} \end{array}$$

*Adsorption Experiment*: It was conducted by mixing 0.2 g of kaolinite with PVOH solutions of varying concentrations (10–80 mg L^–1^) in sealed Erlenmeyer flasks. Samples were equilibrated for 10 h at 150 rpm using an orbital shaker (Sanyo Orbit, Japan) at 298 ± 1 K. Suspensions were subsequently centrifuged (OSK12710, Gawasieki Co., Japan) for 30 min. The amount of PVOH adsorbed at equilibrium (*q*_e_, mg g^−1^) was calculated using the mass‐balance equation (Equation 2), where *C*_0_ and *C*_e_ are the initial and equilibrium PVOH concentrations (mg L^−1^), respectively, *V* is the solution volume (L), and *m* is the dry mass of kaolinite (g).

(2) $$q_{e}=\frac{\left(C_{0}-C_{e}\right)V}{m}$$

To assess possible PVOH precipitation during centrifugation, PVOH‐only control solutions were subjected to the same centrifugation conditions without kaolinite. No measurable change in the PVOH concentration was observed before and after centrifugation, indicating that PVOH remained in solution. The influence of pH on adsorption was investigated over the range of 2–10 using HNO_3_ and KOH solutions to adjust pH. Electrolyte effects were studied by preparing polymer solutions containing NaCl concentrations ranging from 0.01 to 0.5 M. The influence of ultrasonication on adsorption behavior was evaluated using sonicated kaolinite samples. All experiments were performed in triplicate and reported as the mean ± standard deviation.

*Adsorption Isotherm and Layer Thickness Analysis*: Adsorption equilibrium data were analyzed using the Langmuir and the Freundlich adsorption models. The nonlinear form of the Langmuir isotherm is expressed as Equation (3) and the linearized form of the Langmuir equation is Equation (4), where *q*_max_ (mg g^–‍1^) denotes the maximum adsorption capacity and *K*_L_ (L mg^–1^) is the Langmuir adsorption constant. The Freundlich isotherm model is expressed as Equation (5), and its linear form can be written as Equation (6), where *k*_f_ is the Freundlich constant and 1/n represents adsorption intensity. Values of 0.1<1/n<0.5 indicate favorable adsorption. The adsorbed layer thickness (*δ*) was calculated according to Equation (7), where Γ is the adsorption density (mg m^–2^) and *ρ* represents polymer density.

(3) $$q_{e}=\frac{q_{\max}k_{L}C_{e}}{1+K_{L}C_{e}}$$

(4) $$\frac{1}{q_{e}}=\frac{1}{k_{L}q_{\max}}\frac{1}{C_{e}}+\frac{1}{q_{\max}}$$

(5) $$q_{e}=k_{f}C_{e}^{1/n}$$

(6) $$\log\;q_{e}=\log\;k_{f}+\frac{1}{n}\log\;C_{e}$$

(7) $$\delta =\frac{\text{Γ}}{\rho }$$

*Kinetic Analysis*: It was analyzed using pseudo‐first‐order and pseudo‐second‐order models. The pseudo‐first‐order model is represented as Equation (8), where *k*_1_ (min^–1^) is the first‐order rate constant and *q*_t_ (mg g^–1^) is the adsorption capacity at time *t*. The pseudo‐second‐order kinetic model is expressed in Equation (9), where *k*_2_ (g mg^–1^ min^–1^) is the pseudo‐second‐order rate constant. The most suitable kinetic and adsorption models were selected based on the regression coefficient (*R* ^2^).

(8) $$\ln\;(q_{e}-q_{t})=\ln\;q_{e}-k_{1}t$$

(9) $$\frac{t}{q_{t}}=\frac{1}{k_{2}q_{e}^{2}}+\frac{t}{q_{e}}$$

### 2.3 Characterization and Measurements

Kaolinite morphology was characterized using scanning electron microscopy (SEM, JEOL, Japan). Particle size distribution was measured using a Malvern Instruments, UK. Surface area measurements were performed using an Autosorb‐1 porosity analyzer (Quanta Chrome, USA). Electrophoretic mobility and zeta potential measurements were conducted using a Zetasizer 3000 (Malvern Instruments, Germany) with KCl (0.1 g L^–1^) as background electrolyte after pH equilibration.

PVOH molecular properties were characterized by laser light scattering using a DAWN EOS instrument (Wyatt Technology, USA) with a He–Ne laser source (632.8 nm). FTIR spectra of kaolinite, PVOH, and PVOH‐adsorbed kaolinite samples were obtained using a Tensor FTIR spectrometer (Bruker, Germany) over the range of 4000–400 cm^–1^. The equilibrium concentration of PVOH was determined using a Lambda 800 UV–vis spectrophotometer (PerkinElmer, USA) at a wavelength of *λ*_max_ = 198 nm [21].

## 3 Results and Discussions

### 3.1 Characterization of Kaolin

*The physicochemical properties of kaolinite* strongly influence its interfacial interactions with dissolved polymeric contaminants and therefore play a critical role in governing adsorption behavior. To establish a structure–property relationship relevant to the adsorption mechanism of soluble plastic waste, kaolinite was characterized in terms of morphology, surface chemistry, particle size distribution, and electrokinetic properties. Although a series of characterizations has been reported in our previous report [22], the initial analysis of kaolinites was presented before their adsorption analysis. The kaolinite sample exhibited a specific surface area of 14.17 ± 0.25 m^2^ g^−1^ and a point of zero charge (PZC) at pH 2.03 ± 0.01. The particle‐size distribution measured by laser diffraction ranged from approximately 2 to 60 nm. The particles possessed a narrow size distribution and predominantly negative surface charge under experimental conditions. These properties suggest favorable colloidal stability and substantial surface availability for adsorption processes.

*The surface morphology and architecture of kaolinite* particles were investigated, and the corresponding micrograph is shown in Figure 1a. It reveals a characteristic lamellar morphology composed of stacked platelet‐like structures with rough basal planes and edge‐to‐face orientations. Such a morphology is typical of kaolinite minerals and reflects their layered 1:1 aluminosilicate crystal structure consisting of alternating tetrahedral silica sheets and octahedral alumina sheets [24]. High‐magnification observations further revealed microislands, broken edges, and multistep surface features, indicating structural heterogeneity and partial crystal imperfections [25]. These morphological irregularities are important because adsorption predominantly occurs at surface defects, exposed edges, and high‐energy sites rather than on ideal basal surfaces. Broken and stepped edges expose additional aluminol (Al–OH) and silanol (Si–OH) functional groups, which may serve as adsorption sites via hydrogen bonding and intermolecular interactions with PVOH chains [26]. Kaolinite particles consist of stacked twin‐layer sheets held together primarily through hydrogen bonding. Consequently, the external surfaces and particle edges possess distinct chemical environments [27]. Basal planes are generally dominated by siloxane and silanol functionalities and often maintain a net negative charge (Figure 1b), whereas edge sites exhibit pH‐dependent protonation and deprotonation behavior [10, 23, 28]. Such surface heterogeneity produces chemically distinct adsorption domains and strongly contributes to adsorption selectivity and polymer–surface interactions.

**FIGURE 1:**
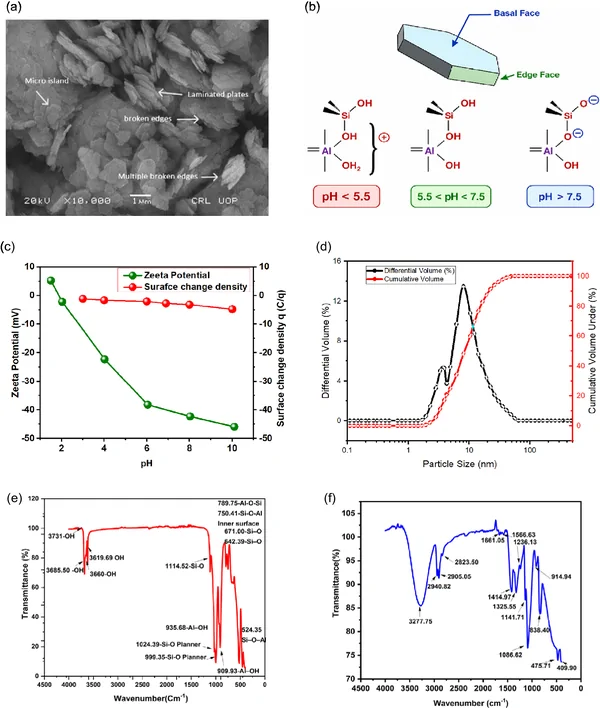
(a) SEM micrograph of kaolinite, (b) the kaolinite and its pH‐dependent surface chemistry (it has been redrawn based on the concept provided by Hocking et al. [23]), (c) pH‐dependent zeta potential and surface charge density, (d) particle size distribution of kaolinite dispersion (1 wt%), (e) FTIR spectrum of kaolinite, and (f) FTIR spectrum of PVOH.

*Electrokinetic behavior* was evaluated by measuring zeta potential as a function of pH, and the results are shown in Figure 1c. The zeta potential is a critical parameter governing colloidal stability because it reflects the electrostatic interactions at the particle–solution interface. Systems possessing high absolute zeta potential values generally exhibit stronger electrostatic repulsion among particles and therefore improved dispersion stability. The zeta potential of kaolinite remained negative throughout the investigated pH range (2–10) and became progressively more negative with increasing pH. The isoelectric point was observed near pH ≈2, in agreement with reported literature values [5, 10, 28]. The increase in negative surface potential at alkaline pH can be attributed to the progressive deprotonation of surface silanol and aluminol groups, generating negatively charged surface sites. As pH increases, greater deprotonation generates additional negative surface sites, increasing electrostatic repulsion among kaolinite particles. Consequently, particle aggregation becomes less favorable, and more stable dispersions are formed. At pH 6, the measured zeta potential reached approximately −39 mV, indicating a highly stable colloidal system. High colloidal stability increases the accessible surface area and facilitates effective interaction between kaolinite and dissolved polymer molecules.

The physicochemical characteristics of the kaolinite are broadly consistent with those reported for kaolin‐based adsorbents. Previous studies have shown that kaolinite exhibits characteristic surface hydroxyl groups and adsorption behavior that depends on surface modification and adsorbate properties. For example, raw and acid‐activated kaolinite showed high methylene‐blue removal and pseudo‐second‐order kinetics, whereas activated natural kaolinite followed the Langmuir model [29, 30, 31]. These findings support the suitability of kaolinite as an adsorbent while highlighting the influence of adsorbate and surface properties on adsorption behavior.

*The variation in surface charge density* with pH, determined by polyelectrolyte titration, is also shown in Figure 1c. Surface charge density increased gradually as pH increased, consistent with electrokinetic measurements. The observed trend can be explained by the anisotropic charge distribution of kaolinite surfaces. Kaolinite particles possess permanently charged basal surfaces together with pH‐responsive edge sites. Under acidic conditions, protonation of surface hydroxyl groups reduces the negative surface charge and can even induce a partial positive charge in edge regions. As pH increases, deprotonation becomes dominant, increasing the density of negatively charged functional groups. The surface charge density reached approximately −2.0 ± 0.10 C g^−1^ at pH 6, indicating significant charge accumulation at the particle–water interface [32]. Surface charge density directly influences electrostatic interactions, polymer configuration near interfaces, and adsorption behavior. Therefore, changes in surface charge are expected to affect both adsorption capacity and adsorption mechanism.

*The particle size distribution of kaolinite* dispersions prepared by magnetic homogenization is shown in Figure 1d. The dispersion exhibited a bimodal distribution, with particle sizes confined entirely within the nanoscale range between approximately 2 and 60 nm. Analysis of the cumulative volume under (%) the curve reveals a sharp, well‐defined transition in the nanoscale regime. The specific percentile metrics extracted from the cumulative distribution show that 10% of the cumulative volume (D_10_) lies below 2.99 nm, the median diameter (D_50_) is reached at 7.91 nm, where 50% of the volume is accounted for, and 90% of the cumulative volume (D_90_) falls below 21.35 nm, yielding a narrow distribution span of 2.32. To validate these findings, the structural properties were cross‐examined using X‐ray diffraction (XRD) data. Calculation via the Scherrer equation yielded an average crystallite size of 14 nm. This result strongly correlates with the nanoscale distribution profile observed in the graphic plot, confirming that the material consists exclusively of true nanoparticles without micron‐scale agglomeration.

The occurrence of bimodal particle populations suggests the coexistence of individual particles and weakly associated aggregates. Such behavior is expected for layered clay systems because interparticle hydrogen bonding and electrostatic interactions can promote partial agglomeration [33]. Particle size is a crucial factor affecting adsorption because smaller particles provide a higher specific surface area and greater availability of adsorption sites. A narrow distribution also indicates relatively homogeneous dispersion behavior and contributes to reproducible adsorption performance.

*The FTIR spectrum of kaolinite* is presented in Figure 1e and provides important information regarding structural functional groups and surface chemistry relevant to adsorption processes. The absorption bands at 3731, 3685.50, and 3660 cm^−1^ correspond to stretching vibrations of external hydroxyl groups, whereas the band observed at 3619 cm^−1^ is attributed to inner hydroxyl groups associated with the octahedral layer [34]. The presence of these hydroxyl groups confirms the abundance of surface‐active functionalities capable of engaging in intermolecular interactions [35]. The bands observed at 935.68 and 909.93 cm^−1^ are assigned to the inner‐surface Al–OH bending mode (hydroxyl deformation modes). The clear separation and high resolution of the 935.68/909.93 cm^−1^ doublet serve as a definitive benchmark confirming the preservation of well‐coordinated dioctahedral layers. Peaks at 1114, 1024, and 999.35 cm^−1^ are associated with Si–O stretching vibrations, whereas bands near 789 and 750 cm^−1^ correspond to Al–O–Si vibrations. This doublet (789.75 and 750 cm^−1^) is assigned to the coupled Si–O–Al stretching and bending structural lattice translations, which are associated directly with stable, high‐temperature‐resilient structural frameworks [36]. The peak at 671.00 cm^−1^ represents the out‐of‐plane bending mode of the Si–O framework. A sharp absorption band at 524.35 cm^−1^ corresponds directly to the internal Si–O–Al bending vibration, confirming that the sheet‐to‐sheet bridging layer remains robustly bound. The observed peak positions agree closely with reported kaolinite spectra [34, 35, 36, 37]. The abundance of surface hydroxyl groups is particularly important for the adsorption of PVOH because these groups can form extensive intermolecular hydrogen‐bonding networks with hydroxyl‐rich polymer chains. Therefore, the FTIR results support the hypothesis that hydrogen bonding, in addition to van der Waals interactions, contributes significantly to the adsorption mechanism [38].

### 3.2 Polymer Characterization

*The molecular characteristics of PVOH* were investigated using laser light scattering to confirm the molecular‐weight distribution. It confirmed the presence of four distinct molecular‐weight fractions at 49, 72, 96, and 125 kDa, designated PVOH_1_, PVOH_2_, PVOH_3_, and PVOH_4_, respectively. Molecular mass is a key physicochemical parameter governing polymer adsorption because it directly influences chain length, hydrodynamic volume, segmental mobility, and the density of adsorption‐active functional groups available for interaction with the kaolinite surface [1]. Increasing the molecular mass generally results in larger polymer coil dimensions and enhanced interfacial contact with solid surfaces, thereby promoting stronger adsorption interactions and greater adsorption capacities [1, 5]. Higher‐molecular‐weight polymers also possess greater conformational flexibility and multiple anchoring points that can interact simultaneously with adsorption‐active sites on the mineral surface [15]. Consequently, characterizing the molecular‐weight distribution is essential for understanding the adsorption mechanism and the architecture of the adsorbed PVOH layer on kaolinite surfaces.

*The FTIR spectrum of PVOH*, shown in Figure 1f, provides important insight into the functional groups responsible for polymer–surface interactions. A broad and intense absorption band centered at approximately 3277.75 cm^–1^ corresponds to O–H stretching vibrations associated with extensive intermolecular and intramolecular hydrogen bonding within the polymer network. The pronounced broadness of this peak confirms the hydroxyl‐rich nature of PVOH and indicates strong hydrogen‐bond interactions among polymer chains. The absorption bands at 2940 and 2905 cm^–1^ are attributed to asymmetric and symmetric stretching vibrations of methylene (–CH_2_–) groups, confirming the polymer’s hydrocarbon backbone [39]. The band located at 1414 cm^–1^ arises from combined C–H bending and O–H deformation vibrations, whereas the prominent peak near 1141 cm^–‍1^ corresponds to C–O stretching vibrations of alcohol groups within the polymer backbone [21, 39]. These hydroxyl‐containing functional groups are expected to play a dominant role in adsorption through hydrogen bonding with surface silanol and aluminol groups present on kaolinite. Collectively, the spectroscopic results confirm the chemical structure of PVOH and strongly support hydrogen bonding and van der Waals interactions as the primary driving forces governing polymer adsorption onto kaolinite surfaces.

### 3.3 Adsorption of PVOH on Kaolinite

*The adsorption behavior* of PVOH on kaolinite was investigated using the Langmuir and the Freundlich adsorption isotherm models, and the corresponding plots are presented in Figure ‍2a,b. For all molecular‐weight fractions of PVOH, adsorption increased rapidly at lower equilibrium concentrations and gradually approached a plateau at higher concentrations, indicating progressive saturation of the available adsorption‐active sites on the kaolinite surface [40]. Initially, the abundance of free surface sites facilitated strong polymer–surface interactions and rapid adsorption; however, as adsorption proceeded, occupation of these sites reduced the number of accessible binding locations, leading to slower adsorption rates and eventual equilibrium [21, 41]. The adsorption capacity increased with both equilibrium concentration and molecular mass of PVOH, suggesting that larger polymer chains possess a stronger affinity toward the kaolinite surface due to their greater chain length and higher density of hydroxyl‐containing repeating units capable of forming intermolecular interactions with surface silanol and aluminol groups. Among the models examined, the Langmuir model provided the best fit to the experimental data, with a regression coefficient (*R* ^2^) of 0.99, compared with approximately 0.93 for the Freundlich model (Table 1). This result indicates that adsorption predominantly occurred through monolayer formation on relatively homogeneous adsorption sites [29, 42]. Furthermore, the calculated separation factor (RL) values were all lower than unity, confirming favorable adsorption behavior. The predominance of Langmuir‐type adsorption suggests that localized surface interactions, particularly hydrogen bonding and van der Waals forces, govern the adsorption process rather than multilayer accumulation under the experimental conditions investigated [15, 40, 41, 42].

**FIGURE 2:**
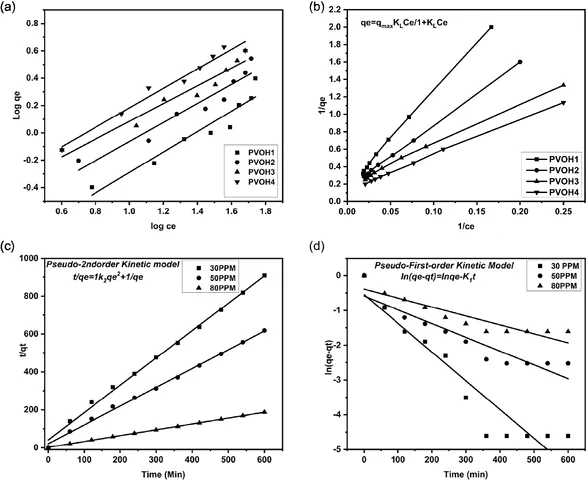
(a) Langmuir adsorption isotherms; (b) Freundlich adsorption isotherms; (c) pseudo‐second‐order kinetic plots; and (d) pseudo‐first‐order kinetic plots for the adsorption of PVOH on kaolinite.

**TABLE 1:** Adsorption isotherm parameters for PVOH on kaolinite.

Sample	Langmuir					Freundlich		
	***q***_**max**_ **, mg g** ^**–‍1**^	**KL, L mg** ^**–‍1**^	RL	***R*** ^**2**^		KF	1/*n*	***R*** ^**2**^
PVOH1	6.55	0.0132	0.602	0.9934		0.0917	0.748	0.9005
PVOH2	6.65	0.0208	0.491	0.9889		0.1733	0.697	0.9082
PVOH3	6.93	0.0388	0.340	0.9940		0.2716	0.650	0.9387
PVOH4	7.92	0.0311	0.392	0.9987		0.2946	0.713	0.9362

*Note* *:* *q*_max_ – maximum monolayer adsorption capacity; KL – Langmuir adsorption constant; RL – dimensionless separation factor; KF – Freundlich adsorption constant; 1/*n* – adsorption intensity parameter; *R* ^2^ – regression coefficient.

To evaluate the mass‐transfer mechanisms and rate‐controlling steps governing the adsorption process, the experimental data were analyzed using pseudo‐first‐order and pseudo‐second‐order kinetic models (Figure 2c,d). For all molecular‐weight fractions of PVOH, a rapid initial adsorption rate was observed during the first few hours, followed by a gradual deceleration until an equilibrium plateau was established. This initial rapid uptake is attributed to the high concentration gradient and the abundance of unoccupied active silanol and aluminol sites on the kaolinite surface.

Among the kinetic models examined, the pseudo‐second‐order model (Figure 2c) provided a better fit to the experimental data, yielding *R* ^2^ > 0.99584 compared with *R* ^2^ ≈ 0.8848 for the pseudo‐first‐order model (Figure 2d). The calculated equilibrium adsorption capacities (*q*_e_,cal) from the pseudo‐second‐order model were also in good agreement with the experimentally obtained values (*q*_e_,exp). These results indicate that the pseudo‐second‐order model describes the observed adsorption kinetics well; however, the model fit alone does not establish a specific adsorption mechanism. Other kinetic and isotherm models may provide additional insight and could be examined in future studies.

*The influence of the polymer molecular mass* on adsorption capacity is presented in Figure 3a. The adsorption capacity increased progressively with increasing logarithm of molecular mass, indicating a strong dependence of adsorption behavior on polymer chain length. Among the investigated samples, PVOH1 exhibited the lowest adsorption capacity (~2.5 mg g^–1^), whereas PVOH4 showed the highest adsorption (~4.5 mg g^–1^) [15, 21, 25]. This trend can be attributed to the increased number of repeating hydroxyl‐containing monomer units in higher‐molecular‐weight polymers. Longer polymer chains provide more adsorption‐active functional groups and larger contact areas with the kaolinite surface, thereby enhancing intermolecular interactions. Higher‐molecular‐mass polymers also possess larger hydrodynamic radii and greater conformational flexibility, enabling the simultaneous formation of multiple surface attachment points. Such multipoint attachment stabilizes adsorption and promotes stronger polymer anchoring on the mineral surface [15, 21, 42]. In addition, larger polymer coils may extend further into solution while remaining attached to the surface through loops and tails, thereby increasing the adsorbed mass per unit surface area. The observed molecular‐weight dependence strongly suggests that adsorption is governed not only by surface chemistry but also by polymer chain architecture and conformational behavior in aqueous media [41].

**FIGURE 3:**
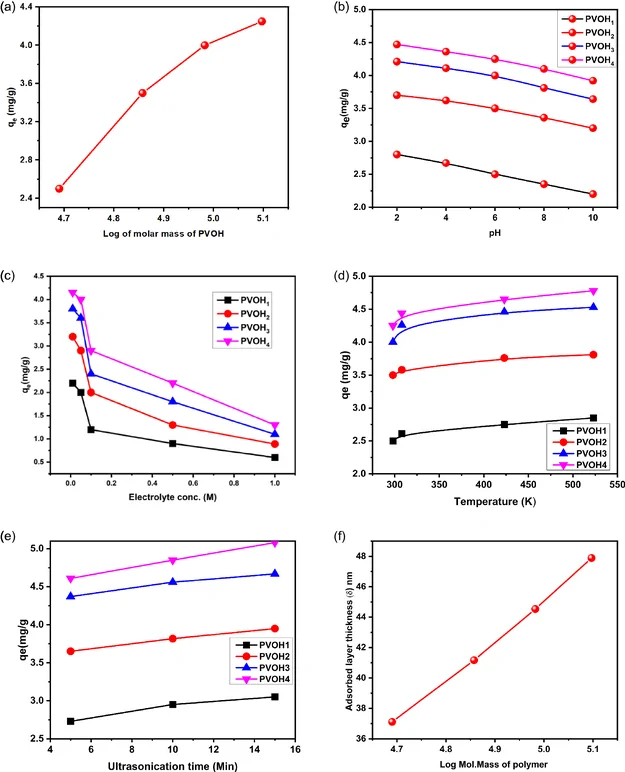
(a) Influence of molecular mass and physicochemical conditions on the adsorption behavior of PVOH on kaolinite: (a) adsorption capacity versus logarithm of PVOH molecular mass, (b) pH effect, (c) electrolyte concentration effect, (d) thermal pretreatment effect, (e) ultrasonication effect, and (f) adsorbed layer thickness as a function of the logarithm of PVOH molecular mass.

The observed increase in maximum adsorption capacity (*q*_max_) with increasing PVOH molecular mass is attributed to differences in macromolecular conformation at the solid–liquid interface. Higher‐molecular‐mass chains can form loops and tails that increase the polymer mass accommodated per unit surface area, while their greater number of hydroxyl groups provides more opportunities for multipoint interactions with kaolinite. To assess possible precipitation, control PVOH solutions were examined under the same experimental conditions, and no visible phase separation or precipitation was observed. These results support surface adsorption rather than bulk precipitation.

*The effect of pH on the adsorption behavior* is illustrated in Figure ‍3b. PVOH adsorption was strongly dependent on solution pH, with maximum adsorption under acidic conditions and progressively decreasing adsorption at higher pH values. Similar adsorption trends were observed for all molecular‐weight fractions, indicating that the influence of pH is largely independent of polymer molecular mass [9, 21, 25]. The strong pH dependence originates primarily from pH‐induced changes in the surface chemistry of kaolinite. As demonstrated earlier through zeta potential and surface charge density measurements, kaolinite surfaces become increasingly negatively charged with increasing pH due to deprotonation of silanol and aluminol groups. Under acidic conditions, partial protonation of edge sites reduces electrostatic repulsion and promotes stronger intermolecular interactions between the polymer and the mineral surface [21, 25]. Hydrogen bonding plays a dominant role in adsorption at lower pH values because both kaolinite hydroxyl groups and PVOH hydroxyl groups remain favorably protonated for intermolecular association. As pH increases, increased negative charge density on the kaolinite surface enhances electrostatic repulsion and weakens adsorption interactions. Moreover, changes in solvent quality and polymer conformation at higher pH may reduce the effective adsorption affinity of PVOH. The decrease in adsorption with increasing pH, therefore, results from the combined effects of electrostatic repulsion, altered surface charge density, and changes in polymer conformation in solution.

*The effect of electrolyte concentration* on PVOH adsorption was investigated using NaCl concentrations ranging from 0.01 to 0.5 M, and the results are presented in Figure 3c. The adsorption capacity decreased progressively with increasing electrolyte concentration, with the most pronounced reduction observed at the highest salt concentrations. Electrolytes strongly influence adsorption processes by modifying the electrostatic environment surrounding both the polymer molecules and the kaolinite surface [15, 42]. The presence of dissolved ions compresses the electrical double layer and screens electrostatic interactions between the adsorbent and adsorbate. Although PVOH is essentially nonionic, adsorption still depends significantly on hydrogen bonding and localized electrostatic interactions involving surface hydroxyl groups [41, 42, 43]. At elevated electrolyte concentrations, competition between electrolyte ions and polymer molecules for adsorption‐active sites may occur. In addition, the “*salting‐out*” effect induced by high ionic strength can alter polymer hydration and reduce effective polymer–surface affinity [15, 43]. Electrolyte ions may also occupy surface‐active hydroxyl sites on kaolinite, thereby decreasing the number of available adsorption locations for PVOH molecules. Consequently, increasing ionic strength weakens polymer–surface interactions and suppresses adsorption efficiency [42, 43, 44].

*The influence of thermal pretreatment* of kaolinite on adsorption is shown in Figure 3d. Kaolinite samples were heated between 298 and 523 K prior to the adsorption experiments. Moderate thermal treatment significantly enhanced the adsorption capacity, with maximum adsorption observed near 393 K. Beyond this temperature, adsorption gradually decreased. The initial enhancement in adsorption can be attributed to the removal of physically adsorbed water and volatile impurities from the kaolinite surface during heating. Desorption of water molecules exposes previously inaccessible adsorption‐active sites and increases surface accessibility [15, 21, 42]. Thermal treatment may additionally improve pore accessibility and surface roughness, thereby enhancing polymer adsorption. However, heating beyond approximately 393 K reduced the adsorption capacity. This decrease may arise from structural alteration or partial dehydroxylation of kaolinite, which reduces the number of surface hydroxyl groups available for hydrogen bonding with PVOH. At elevated temperatures, kaolinite may undergo structural transformation toward amorphous aluminosilicate phases or transition‐metal aluminas, altering surface chemistry and reducing adsorption affinity [15, 21, 25, 44]. The results, therefore, indicate that moderate thermal activation enhances adsorption, whereas excessive thermal treatment adversely affects the surface functionality.

*The influence of ultrasonication* on PVOH adsorption is shown in Figure 3e. Ultrasonication significantly enhanced the adsorption capacity for all polymer fractions. This enhancement directly correlates with the structural and physicochemical modifications induced by cavitation‐driven particle fragmentation. Ultrasonication reduced particle size, increased surface area, improved dispersion stability, and increased surface charge density, all of which collectively favored adsorption [25, 32, 45]. Fragmentation of kaolinite aggregates exposed additional adsorption‐active edge sites and increased accessibility of surface hydroxyl groups capable of interacting with PVOH. The enhanced colloidal stability generated by ultrasonication also reduced particle agglomeration and promoted a more uniform polymer distribution within the dispersion [46]. In the presence of polymer molecules, deagglomerated particles remained suspended for longer durations, thereby increasing the polymer–surface contact time and improving adsorption efficiency. Furthermore, adsorption increased systematically with increasing PVOH molecular mass, indicating that larger polymer chains benefited more strongly from the increased surface accessibility generated by ultrasonication [15, 21, 24, 25].

*The adsorbed layer thickness* increased progressively with increasing adsorption density and polymer molecular mass (Figure 3f). Initially, *δ* increased gradually, whereas a more pronounced increase occurred at higher adsorption densities. This behavior suggests the development of extended polymer conformations and the possible formation of multilayer‐like surface structures at higher surface coverage [46, 47]. The increase in adsorbed layer thickness with molecular mass indicates that longer polymer chains extend further from the kaolinite surface into the surrounding solution phase. Such a behavior is characteristic of polymer adsorption structures composed of trains, loops, and tails. The adsorbed polymer chains are therefore not completely flattened on the surface but instead adopt partially extended conformations [48]. These findings are consistent with established polymer adsorption theories and previously reported adsorption behavior of hydroxyl‐containing polymers on clay mineral surfaces [46, 47, 48].

### 3.4 Mechanism of PVOH Adsorption on Kaolinite

Previous studies have also demonstrated that kaolinite can effectively interact with large carbon‐based organic structures, including humic acid, supporting its capacity to accommodate organic macromolecules on its surface [49]. The adsorption process was further examined using FTIR spectroscopy by comparing pure kaolinite, pure PVOH, and PVOH‐treated kaolinite (Figure 4a). The kaolinite spectrum showed characteristic Al–OH stretching bands at 3694, 3662, and 3623 cm^−1^ [50]. These bands were retained after PVOH treatment, indicating that the kaolinite structure was preserved. The PVOH‐treated kaolinite also exhibited characteristic polymer bands, including C–H stretching near 2885 cm^−1^ and C–O‐related absorption near 1116 cm^−1^. These spectral features are consistent with the presence of PVOH and interactions between PVOH functional groups and kaolinite surface functionalities. However, FTIR spectroscopy alone cannot distinguish surface adsorption from physical association; therefore, the FTIR results are interpreted as complementary evidence alongside the adsorption measurements and centrifugation controls.

**FIGURE 4:**
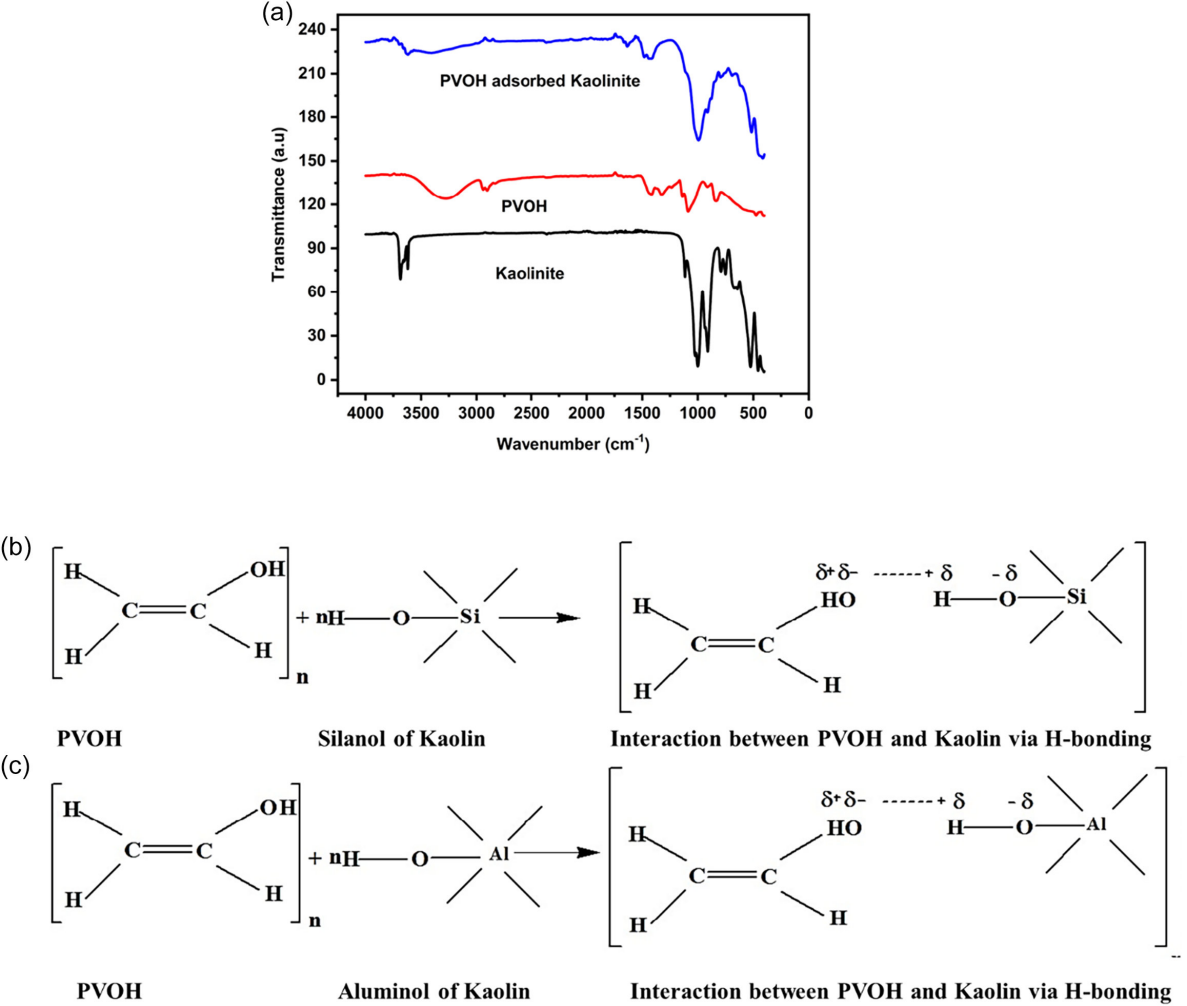
(a) FTIR spectra of kaolinite, PVOH, and PVOH‐treated kaolinite; (b) proposed PVOH–kaolinite interaction via silanol groups; and (c) proposed interaction via aluminol groups.

The combined physicochemical and spectroscopic results support the contribution of hydrogen bonding, van der Waals interactions, and weak electrostatic interactions to PVOH–kaolinite interactions (Figure 4b,c). The adsorption of PVOH exhibited moderate pH dependence, with adsorption capacity decreasing by approximately 18% as pH increased from 2 to 10 [48, 51, 52]. This behavior is closely associated with the pH‐dependent surface charge characteristics of kaolinite, as confirmed by the zeta potential and surface charge density measurements (Figure 1c). At acidic pH, protonation of surface hydroxyl groups reduces the negative surface charge of kaolinite and promotes intermolecular interactions between PVOH and the mineral surface [52, 53]. Conversely, increasing pH enhances deprotonation of silanol and aluminol groups, generating a more negatively charged surface that weakens adsorption through electrostatic repulsion and altered polymer conformation. However, the relatively limited reduction in adsorption across the investigated pH range indicates that electrostatic interactions are not the dominant adsorption force [54]. Ultrasonication further enhanced adsorption by generating new active surfaces and exposing additional surface cations, such as Al^3+^ and Mg^2+^, which may serve as bridging centers between the polymer and the kaolinite surface [53, 54, 55]. These hydrated cations also facilitate intermolecular hydrogen bonding by interacting strongly with water molecules. Furthermore, increasing electrolyte concentration significantly suppressed adsorption due to competitive occupation of adsorption‐active sites by Na^+^ and Cl^−^ ions and compression of the electrical double layer, thereby reducing polymer–surface interactions. Higher‐molecular‐weight PVOH fractions exhibited greater adsorption capacities because longer polymer chains have larger hydrodynamic dimensions and more hydroxyl‐containing repeating units that can form multiple surface attachment points simultaneously [56].

## 4 Conclusion

This study demonstrates that the adsorption of PVOH onto kaolinite is strongly governed by the polymer’s molecular structure and the physicochemical properties of the clay surface. Adsorption capacity increased with polymer molecular mass and initial concentration owing to enhanced surface interactions and improved utilization of available adsorption sites. Langmuir adsorption behavior (*R* ^2^ > 0.99) confirmed monolayer adsorption on relatively homogeneous surfaces. Kinetic modeling demonstrated that the adsorption process followed a pseudo‐second‐order kinetic profile rather than a pseudo‐first‐order mechanism. This behavior confirms that the rate‐controlling step is governed by strong localized chemical or physical interactions, primarily hydrogen bonding and van der Waals forces between the hydroxyl groups of the PVOH chains and the active sites on the kaolinite surface, rather than being controlled by mass transport or film diffusion. Adsorption decreased by approximately 18% as pH increased owing to enhanced negative surface charge and electrostatic repulsion, whereas elevated electrolyte concentrations reduced adsorption through competitive ionic interactions and active‐site shielding. Thermal pretreatment and ultrasonication significantly improved adsorption by increasing surface area, surface charge density, and accessibility of reactive adsorption sites. FTIR and physicochemical analyses confirmed that adsorption was predominantly governed by hydrogen bonding between hydroxyl groups of PVOH and silanol/aluminol groups of kaolinite, with van der Waals forces providing additional stabilization. The findings establish modified kaolinite as a low‐cost, environmentally sustainable, and scalable adsorbent for remediating soluble plastic contaminants in wastewater and landfill systems.

## Funding

This work was supported by the Deanship of Scientific Research at King Khalid University for supporting this work through the Research Group Program (RGP‐2/518/46). Dr. Mahmud was not involved in this grant.

## Ethics Statement

This article does not contain any studies with human participants or animals performed by any of the authors.

## Conflicts of Interest

The authors declare no conflicts of interest.

## Data Availability

The authors confirmed that the data supporting the findings of this study are available within the article.
